# The Short‐Term Versus Long‐Term Effects of Acceptance and Moderation by Stimulus Arousal Level

**DOI:** 10.1111/psyp.70410

**Published:** 2026-09-25

**Authors:** Nicole R. Baughman, Annmarie MacNamara

**Affiliations:** ^1^ Department of Psychological and Brain Sciences Texas A&M University College Station Texas USA; ^2^ Department of Psychiatry and Behavioral Sciences Texas A&M University College Station Texas USA; ^3^ Institute for Neuroscience Texas A&M University College Station Texas USA

**Keywords:** acceptance, arousal, emotion regulation, engagement‐disengagement framework, event‐related potential (ERP), late positive potential (LPP)

## Abstract

Negative emotion down‐regulation strategies vary in the depth of emotional engagement they entail, which may lead to different short‐ versus longer‐term effects. Acceptance is an emotion regulation strategy in which an individual allows emotions to be present without letting them fuse with the self or overwhelm their experience. Acceptance involves a relatively deep level of emotional processing but has been understudied compared to other engaged strategies. In particular, it is unknown whether acceptance is subject to the same moderators (e.g., stimulus arousal level) and how this might vary in the short versus longer term. Here, participants (*n* = 79) accepted or viewed low‐ and high‐arousing negative pictures. Immediately after, a subset of participants (*n* = 33) viewed the same pictures, without instructions to regulate. In the first task, acceptance reduced the late positive potential (LPP) to low‐, but not high‐arousing pictures only early on during picture presentation. Additionally, acceptance reduced arousal ratings for both low‐ and high‐arousing pictures. In the second task, previously accepted low‐ and high‐arousing pictures elicited smaller LPPs than previously viewed pictures, with this effect emerging more quickly for low‐arousing pictures. Arousal and valence ratings did not differ between previously accepted versus viewed pictures. Therefore, although acceptance may be less effective at reducing the motivational salience of high‐arousing stimuli in the short term, it leads to longer‐term reductions in electrocortical processing of both low‐ and high‐arousing pictures. Results extend the engagement‐disengagement framework to include acceptance, suggesting that it shares strengths and weaknesses with other engaged emotion regulation strategies.

## Impact Statement

Acceptance has been relatively understudied, limiting understanding of its utility, its moderators and its place within the engagement‐disengagement framework. Here we show that acceptance is less effective for highly arousing stimuli in the short term but leads to lasting reductions in electrocortical responding to both low‐ and high‐arousing stimuli when pictures are encountered later, outside of an emotion regulation context. Therefore, acceptance appears to share strengths and weaknesses with other engaged negative emotion down‐regulation strategies.

## 1 Introduction

Emotion regulation strategies vary in the depth of emotional processing they entail. Disengaged strategies (e.g., thinking unrelated thoughts) involve minimal processing of stimulus content whereas engaged strategies (e.g., reinterpreting meaning) involve much deeper processing. Both engaged and disengaged strategies are effective at reducing response to negative stimuli; however, these strategies have unique strengths and limitations and may produce different outcomes in the short versus long term (MacNamara 2025; Sheppes and Meiran 2007, 2008). Although it has been proposed that engaged negative emotion regulation strategies should resemble each other in terms of moderators and short‐ versus long‐term effects (MacNamara 2025), few studies have examined engaged strategies other than reappraisal. Acceptance is an engaged emotion regulation strategy that entails allowing emotions to be present, while emphasizing that they do not need to fuse with the self or dominate a person's experience (Hayes et al. 1999; Keogh et al. 2005; MacNamara 2025). Prior work has shown that acceptance can decrease self‐reported negative emotion as well as neural and physiological correlates of negative emotional responding (Goldin et al. 2019; Kross et al. 2009; Segal et al. 2025). Nonetheless, little is known about the factors that might modulate acceptance's success or its short‐ versus long‐term effects, leaving a critical hole in the engagement‐disengagement framework and little knowledge of the situations to which acceptance might be best suited (MacNamara 2025).

Event‐related potentials are a useful tool for measuring the effects of both engaged and disengaged emotion regulation strategies. For instance, the late positive potential (LPP) is a positive‐going, centroparietally maximal event‐related potential that begins approximately 400 ms after stimulus onset and persists through the duration of stimulus presentation (Cuthbert et al. 2000). It is larger for emotionally arousing stimuli compared to non‐emotional stimuli and is sensitive to willful attempts to alter emotional responding (Hajcak et al. 2010; MacNamara et al. 2022). For example, cognitive reappraisal and distraction can reduce the LPP elicited by emotional pictures (Hajcak and Nieuwenhuis 2006; MacNamara et al. 2009; Shafir et al. 2016; Thiruchselvam et al. 2011). The LPP has also been used to examine factors that moderate the success of engaged versus disengaged emotion regulation strategies, and to assess their relative strengths and weaknesses (Boehme et al. 2019; Langeslag and Surti 2017; MacNamara 2025; Schönfelder et al. 2014; Thiruchselvam et al. 2011).

More specifically, although reappraisal can reduce electrocortical responding and subjective ratings of emotional intensity in response to negative stimuli (Foti and Hajcak 2008; Hajcak and Nieuwenhuis 2006; Moser et al. 2006, 2009), numerous studies have found that attempts at emotional down‐regulation via reappraisal failed to reduce, or even increased the LPP (Cao et al. 2020; Gan et al. 2015; Gardener et al. 2013; Imburgio and MacNamara 2019; Sarlo et al. 2013). This may be because reappraisal involves attending to the emotional details of a stimulus prior to generating an alternative stimulus meaning (Strauss et al. 2016), which could cause elevations in emotional responding, at least initially. By contrast, distraction often leads to faster and more pronounced reductions in electrocortical responding to negative stimuli (Schönfelder et al. 2014; Thiruchselvam et al. 2011), suggesting that strategies that involve more intensive engagement with emotion (e.g., reappraisal, acceptance) may be better suited to situations in which someone has the time and resources available to dedicate to sustained processing of emotion.

Because engaged emotion down‐regulation strategies involve deeper processing of stimuli, they tend to be less effective for highly arousing stimuli. For instance, participants report that low‐arousing pictures are easier to reappraise—and reappraisal has been shown to *increase* the LPP elicited for highly arousing stimuli in particular (Langeslag and Surti 2017). Similarly, distraction led to stronger decreases in subjective negative experience and faster reductions in the LPP compared to reappraisal (Shafir et al. 2015). Therefore, the short‐term efficacy of engaged emotion regulation strategies appears to be moderated by stimulus arousal level. That is, disengaged strategies reduce emotional arousal quickly without requiring deeper emotional processing and may therefore be effective for highly arousing stimuli. On the other hand, engaged strategies appear to be more difficult and slower to implement, and may therefore be less effective—in the short term—for high‐arousing stimuli.

Notably, the short‐term effects of emotion regulation strategies are not the only effects of interest, especially considering that in everyday life, people often encounter the same or similar negative situations more than once. Studies have found that reappraisal can reduce long‐term emotional responding to stimuli (Ahn et al. 2015; Hermann et al. 2017, 2021). For example, in the absence of instructions to regulate emotion, negative and neutral pictures that had been previously reappraised roughly 30 min prior elicited smaller LPPs and less arousing picture ratings (MacNamara et al. 2011). Conversely, disengaged emotion regulation strategies may lead to enhanced electrocortical processing of negative stimuli upon subsequent encounters (Jiang et al. 2020; Thiruchselvam et al. 2011). During a passive picture viewing task, unpleasant pictures with a distraction history elicited larger LPPs as compared to pictures that participants had previously attended to (Paul et al. 2016). Therefore, although engaged strategies may be more difficult to implement in the short term, especially for highly arousing stimuli, emotional engagement may be necessary to achieve long‐term reductions in negative emotional responding.

Because acceptance involves recognizing, tolerating and “sitting with” emotional experience, it should share some of the same strengths and limitations as reappraisal—the most well‐studied engaged approach (MacNamara 2025). Indeed, preliminary findings suggest that acceptance may function similarly to other engaged strategies. In a study which labeled the emotion regulation strategy as mindfulness, but which appeared to involve acceptance (i.e., participants were told to “pay attention to all arising thoughts, feelings, and bodily sensations in an accepting manner”), Uusberg et al. (2016) initially observed an increase in the LPP compared to attending to picture details or distraction. Notably, upon re‐exposure to the same pictures without instructions to regulate, amplification of the LPP for negative versus neutral pictures remained present for the other conditions, but not acceptance. In other words, although acceptance had initially increased the LPP, it led to lasting reductions in stimulus processing when pictures were encountered later on.

Similarly, Boehme et al. (2019) also found that acceptance increased the LPP compared to simply viewing anxiety‐inducing pictures. Additionally, acceptance did not reduce subjective ratings of negative emotional experience, though it did reduce skin conductance response. In sum, like reappraisal, acceptance may initially increase emotional responding as a result of increased engagement with emotional experience. However, it is unclear whether the long‐term effects of acceptance are influenced by stimulus arousal. If acceptance increases emotional processing in the short term, especially in response to high‐arousing stimuli, but gives way to longer‐term reductions in negative emotional responding for both high‐ and low‐arousing stimuli, this could have important implications for strategy selection/situation‐matching (Aldao 2013) and would further validate the engagement‐disengagement framework (MacNamara 2025).

Here, we sought to determine whether stimulus arousal would moderate the short‐ and long‐term efficacy of acceptance. Drawing on previous work indicating that reappraisal may be less effective for highly arousing stimuli, at least in the short term (Langeslag and Surti 2017; Shafir et al. 2015), we expected that acceptance would initially increase the LPP (Boehme et al. 2019; Uusberg et al. 2016) particularly for high‐arousing pictures. Upon re‐exposure to pictures without instructions to regulate, we hypothesized that previously accepted pictures would elicit smaller LPPs as well as reduced unpleasantness and arousal ratings compared to previously viewed pictures, regardless of picture arousal level (Ahn et al. 2015; Hermann et al. 2017, 2021; Langeslag and Surti 2017; MacNamara et al. 2011).

## 2 Method

### 2.1 Participants

Eighty‐five participants initially consented for the study. Due to a programming error, data from only 34 individuals were available for the second task (passive picture viewing). In the first task, data from six individuals were excluded due to poor quality recordings (i.e., less than 50% of trials remaining per condition after artifact rejection). In the second task (passive picture viewing), data from one participant were excluded for poor quality recording. Therefore, in total, data were analyzed for 79 participants in the first task (51 female; *M* = 19.23 years, SD = 1.4) and 33 of these participants were included in analysis of the second task (18 female; *M* = 19.52 years, SD = 1.7). For the sake of completeness, analyses were also conducted for the first task including only participants with data available for the passive picture viewing task (*n* = 33)—these analyses are presented in Supporting Information. Study procedures were in compliance with the Helsinki Declaration of 1975 (as revised in 1983) and approved by the Texas A&M Institutional Board.

### 2.2 Materials

Ninety‐six negative pictures (48 high arousing; 48 low arousing) were chosen from the International Affective Picture Systems (IAPS; Lang et al. 1997), Emotional Picture Set (EmoPics; Wessa et al. 2010), and the Open Affective Standardized Image Set (OASIS; Kurdi et al. 2017).^1^ Pictures were chosen from multiple databases to provide increased options for picture selection. Pictures were displayed in color and filled the monitor screen, which measured 48.26 cm diagonally.

Participants used the Self‐Assessment Manikin 9‐point scales (SAM; Bradley and Lang 1994) during the task to rate the valence and arousal of each picture. Response options ranged from 1 to 9, with higher numbers indicating more pleasant valence and more arousing pictures.

### 2.3 Procedure

**FIGURE 1:**
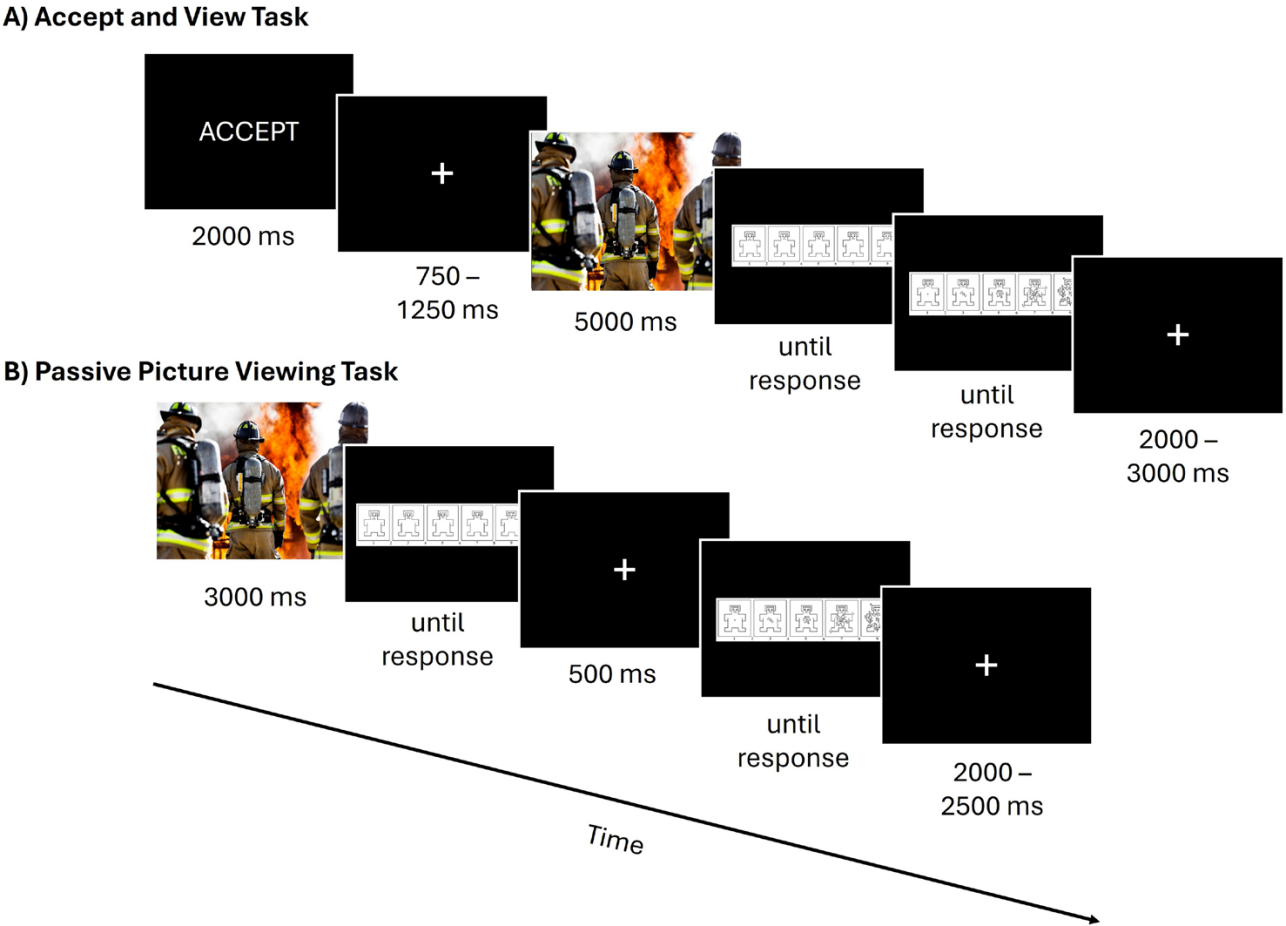
Example trials from (A) the accept and view task and (B) the passive picture viewing task.

Upon arrival, participants were directed to a laboratory room where they provided informed consent and were asked to complete a demographic questionnaire. Participants were trained in acceptance and then completed the Accept and View task. Following the completion of the first task, participants then completed a passive picture viewing task in which they viewed the same pictures that had been presented in the prior task. Figure 1 depicts example trials for both tasks.

#### 2.3.1 Accept and View Task

Participants were told that they would be viewing negative pictures during the task. They were informed that prior to picture onset, they would either see the word “VIEW” or “ACCEPT”. During view trials, participants were asked to view the picture as normal, without trying to alter their emotional response in any way. During accept trials, participants were asked to try to accept the emotions they felt in response to the picture. Participants were trained in acceptance using the following instructions:

In this task, you will be shown negative pictures. On some trials, you will see the word “ACCEPT” before a picture comes onscreen. On these trials, we would like you to shift into an acceptance mind‐frame. What I mean by that is that I'd like you to try to accept your feelings as they arise in response to a picture. But importantly, I'd like you to keep in mind that feelings do not need to control us. To give you a better idea of what I mean, think of your feelings like smoke—you can see, smell and feel smoke—you can name it, you can feel it and it is real. But you don't need to breathe it in and let it suffocate you. We don't need to avoid or deny our feelings, but at the same time, we don't need to fuse with them so that they encompass us completely. An alternative—which we would like you to try to do here—is to accept your feelings for what they are, and to know that they are ok as they are. We want you to keep in mind that it is not necessary to cling to these feelings, try to change or deny them. They can just hang out in space, as they are, and do their thing. We do not need to dwell on them. And we don't need to shut them out.

After receiving task instructions, participants then completed four practice trials during which they practiced accepting negative pictures. To ensure understanding, participants were asked to describe how they used acceptance during the practice trials. If participants needed additional instruction, the experimenter provided examples. After the practice trials, participants practiced using the SAM valence and arousal scales and were informed that they would be asked to rate each negative picture during the task.

During the real experiment, each trial began with the word “VIEW” or “ACCEPT” displayed in white text on a black background for 2000 ms, followed by a fixation cross that varied randomly in duration from 750 to 1250 ms. Then, a negative picture was displayed onscreen for 5000 ms. Following picture offset, participants rated the picture on valence and arousal (self‐paced). Prior to the next trial, a white fixation cross was displayed on a black background for 2000–3000 ms.

There were two “VIEW” blocks and two “ACCEPT” blocks, the order of which were counterbalanced across participants. Picture assignment to condition was randomized for each participant, within the constraint that there were an even number of low and high arousing pictures assigned to each condition. This yielded four possible trial types (view low arousal, view high arousal, accept low arousal, accept high arousal), with six of each trial type presented in each block. Across the task, there were a total of 24 view low arousal trials, 24 view high arousal trials, 24 accept low arousal trials, and 24 accept high arousal trials. Trial order was randomized for each participant, within the constraints noted above. Participants received a self‐paced break between each block.

#### 2.3.2 Passive Picture Viewing Task

Following the Accept and View task, participants completed a passive picture viewing task, in which they were instructed to simply view each of the negative pictures from the previous task, without trying to change their emotional response in any way.

Each trial began with a picture displayed for 3000 ms. Following picture offset, participants rated the picture on valence and arousal (self‐paced), with a 500 ms fixation between each rating. During the intertrial interval, a white fixation cross was displayed on a black background for 2000–2500 ms. The task consisted of three blocks, each containing 32 trials. Across the task, there were a total of 24 previously viewed low arousal pictures, 24 previously viewed high arousal pictures, 24 previously accepted low arousal pictures, and 24 previously accepted high arousal pictures. Trial order was randomized for each participant. Additionally, picture order within each condition was randomized to ensure that pictures were not presented in the same order as the first task within each. Between each block, participants received a self‐paced break.

### 2.4 Electroencephalographic Recording and Data Processing

Continuous EEG was recorded using an ActiCap and the ActiCHamp amplifier system (Brain Products GmbH, Gilching Germany). Thirty‐two electrode sites were used based on the 10/20 system. The electrooculogram (EOG) was recorded from four facial electrodes: two that were placed approximately 1 cm above and below the right eye, forming a bipolar channel to measure vertical eye movement and blinks, and two that were placed approximately 1 cm beyond the outer edges of each eye, forming a bipolar channel to measure horizontal eye movements. The EEG data were digitized at 24‐bit resolution and a sampling rate of 1000 Hz.

EEG data was processed offline using Brain Vision Analyzer 2.3 software (Brain Products GmbH, Gilching Germany). Data from each task were segmented for each trial beginning 200 ms prior to stimulus onset and lasting throughout picture duration. The signal from each electrode was re‐referenced to the average of the left and right mastoids (TP9/10) and band‐pass filtered with high‐pass and low‐pass filters of 0.01 and 30 Hz, respectively. Eye blink and ocular corrections used the method developed by Miller et al. (1988). Artifact analysis was used to identify a voltage step of more than 50.0 μV between sample points, a voltage difference of 300.0 μV within a trial, and a maximum voltage difference of less than 0.50 μV within 100 ms intervals. Trials were also inspected visually for any remaining artifacts, and data from individual channels containing artifacts were rejected on a trial‐to‐trial basis. Amplitudes were averaged separately for each trial type and baseline correction for each trial was performed using the 200 ms pre‐stimulus period. The average percent of trials remaining per condition after artifact rejection was as follows for the Accept and View task: View Low = 90.84%, SD = 11.85, View High = 91.95%, SD = 12.66, Accept Low = 91.70%, SD = 12.98, Accept High = 90.96%, SD = 14.11. The average percent of trials remaining per condition after artifact rejection was as follows for the passive picture viewing task: View Low = 87.46%, SD = 14.00, View High = 86.11%, SD = 16.74, Accept Low = 85.82%, SD = 15.71, Accept High = 86.37%, SD = 13.83.

The LPP was scored at three separate electrodes Fz, Cz, and Pz (Bautista et al. 2022; Langeslag and Surti 2017; Moser et al. 2010). Mean area amplitude was exported between 500–2500 ms and 2500–5000 ms after picture onset in the Accept and View task and between 500–1750 ms and 1750–3000 ms after picture onset in the passive picture viewing task.

### 2.5 Data Analyses

The LPP in each task was analyzed using a 2 (condition: accept, view) × 2 (arousal: high, low) × 3 (electrode: Fz, Cz, Pz) repeated measures analysis of variance (ANOVA), performed separately for each time window. Valence and arousal ratings were analyzed using 2 (condition: accept, view) × 2 (arousal: high, low) repeated measures ANOVAs. Greenhouse–Geisser correction was applied when assumptions of sphericity were violated. Significant effects were followed up using repeated measures ANOVAs and paired *t*‐tests. All analyses were performed using SPSS statistical software version 29.0 (IBM, Armonk, NY).

## 3 Results

### 3.1 Accept and View Task

**FIGURE 2:**
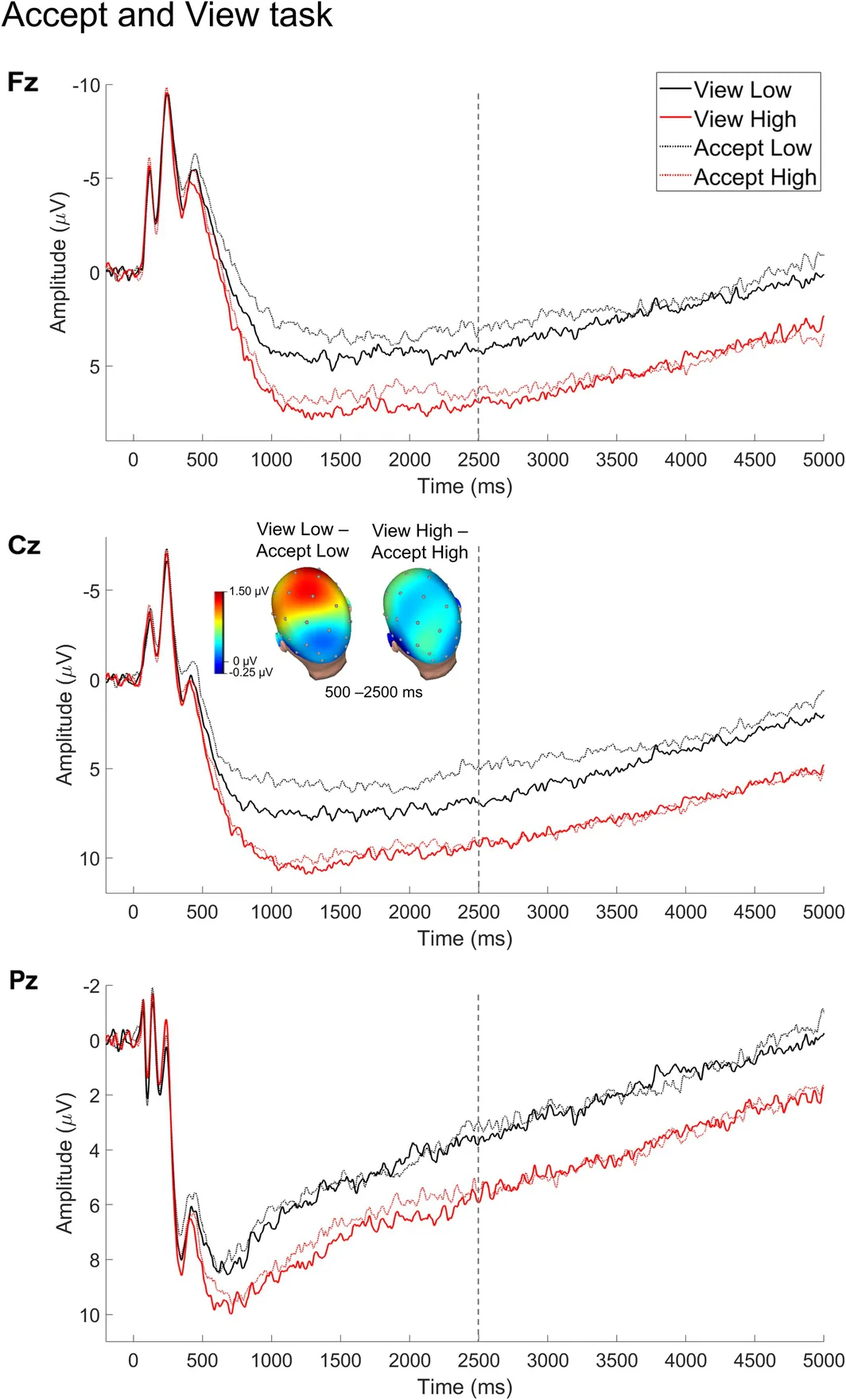
Grand‐averaged waveforms at Fz, Cz, and Pz for the accept and view task shown seprately for low‐ and high‐arousing pictures presented on view and accept trials. “Low” and “High” refer to low‐ and high‐arousing trials, respectively. The dashed line demarcates the border between the early and late time windows in which the LPP was scored. Headmaps depict difference scores for view—accept, shown separately for high‐ and low‐arousing pictures from 500 to 2500 ms after picture onset.

Table 1 presents means and standard deviations for the LPP and picture ratings during the Accept and View task and Figure 2 depicts grand‐averaged waveforms, shown separately for each trial type at electrodes Fz, Cz, and Pz. Headmaps depict the scalp distribution of condition difference scores (view—accept) from 500 to 2500 ms.

**TABLE 1:** Mean (SD) LPP and behavioral ratings separated by condition and arousal during the accept and view task.

Accept and view task				
	View high *M* (SD)	View low *M* (SD)	Accept high *M* (SD)	Accept low *M* (SD)
Fz (μV; 500–2500 ms)	6.12 (5.91)	3.52 (5.07)	5.37 (6.92)	2.32 (5.66)
Cz (μV; 500–2500 ms)	9.52 (5.83)	7.04 (5.02)	9.16 (6.39)	5.48 (5.40)
Pz (μV; 500–2500 ms)	7.58 (5.04)	5.59 (4.49)	7.09 (5.28)	5.44 (4.97)
Fz (μV; 2500–5000 ms)	5.07 (6.58)	2.16 (6.93)	5.05 (8.53)	1.49 (7.22)
Cz (μV; 2500–5000 ms)	7.28 (8.01)	4.48 (7.60)	7.37 (8.10)	3.45 (7.15)
Pz (μV; 2500–5000 ms)	3.76 (7.79)	1.67 (6.58)	3.78 (7.27)	1.55 (6.14)
Valence	3.10 (0.85)	3.92 (0.66)	3.25 (1.01)	4.00 (0.79)
Arousal	3.93 (1.61)	2.70 (1.18)	3.69 (1.52)	2.48 (1.11)

*Note:* Valence and arousal were rated on the 1 to 9 SAM scales with higher numbers indicating more pleasant and arousing ratings. “Low” and “High” refer to low‐ and high‐arousing trials, respectively.

#### 3.1.1 LPP

##### 3.1.1.1 500–2500 ms

As expected, high‐arousing pictures elicited larger LPPs than low‐arousing pictures, *F*(1, 78) = 59.17, *p* < 0.001, *η*_*p*_^2^ = 0.43. In addition, there was a main effect of condition, *F*(1, 78) = 4.27, *p* = 0.04, *η*_*p*_^2^ = 0.05 (View > Accept), which was qualified by a condition × arousal × electrode interaction, *F*(1.65, 128.76) = 4.28, *p =* 0.022, *η*_*p*_^2^ = 0.05. Follow‐up repeated measures ANOVAs revealed a main effect of condition, *F*(1, 78) = 4.30, *p =* 0.04, *η*_*p*_^2^ = 0.05, and a condition × electrode interaction, *F*(1.83, 142.53) = 5.81, *p =* 0.005, *η*_*p*_^2^ = 0.07 for low arousal pictures only. That is, for low arousal pictures, acceptance reduced the LPP at Fz, *t*(78) = 2.17, *p* = 0.03, *d* = 0.24 and Cz, *t*(78) = 2.76, *p* = 0.007, *d* = 0.31. The view versus accept difference did not reach significance at Pz (*p* = 0.739). There were no significant effects involving condition for high arousal pictures (*p*s > 0.25).

##### 3.1.1.2 2500–5000 ms

As expected, high‐arousing pictures elicited larger LPPs than low‐arousing pictures, *F*(1, 78) = 31.43, *p* < 0.001, *η*_*p*_^2^ = 0.29. There were no significant effects involving condition (*p*s > 0.47).

#### 3.1.2 Ratings

High arousal pictures were rated as more arousing than low arousal pictures, *F*(1, 78) = 326.63, *p* < 0.001, *η*_*p*_^2^ = 0.81. There was also a main effect of condition, *F*(1,78) = 6.51, *p =* 0.01, *η*_*p*_^2^ = 0.08, which indicated that acceptance reduced arousal.

For valence ratings, the main effect of arousal was significant, indicating that high arousal pictures were rated as more unpleasant than low arousal pictures, *F*(1, 78) = 163.12, *p* < 0.001, *η*_*p*_^2^ = 0.68. There were no significant effects involving condition (*p*s > 0.12).

### 3.2 Passive Picture Viewing Task

**FIGURE 3:**
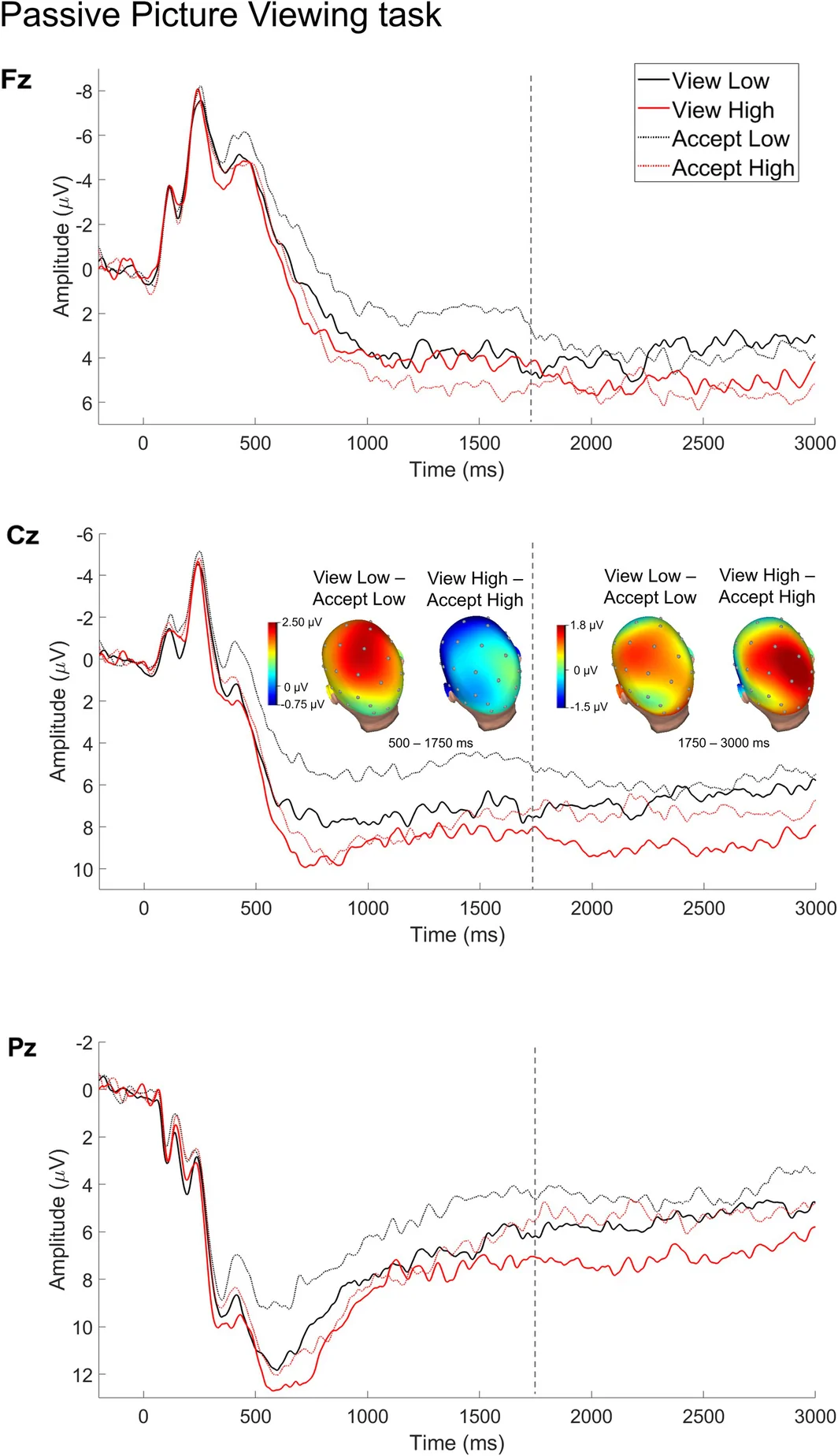
Grand‐averaged waveforms at Fz, Cz and Pz for the passive picture viewing task shown separately for low‐ and high‐arousing pictures presented on view and accept trials. “Low” and “High” refer to low‐ and high‐arousing trials, respectively. The dashed line demarcates the border between the early and late time windows in which the LPP was scored. Headmaps depict difference scores for view—accept, shown separately for high‐ and low‐arousing pictures from 500 to 1750 ms and 1750 to 3000 ms after picture onset. A 12 Hz low‐pass filter was applied to waveforms for data visualization only.

Table 2 presents means and standard deviations for the LPP and picture ratings during the passive picture viewing task, shown separately for each trial type. Figure 3 depicts grand‐averaged waveforms, shown separately for each trial type, at electrodes Fz, Cz, and Pz. Headmaps depict the scalp distribution of condition difference scores (previously viewed—previously accepted) from 500–1750 ms and 1750–3000 ms.

**TABLE 2:** Mean (SD) LPP and behavioral ratings separated by condition and arousal during the passive picture viewing task.

Passive picture viewing task				
	View high *M* (SD)	View low *M* (SD)	Accept high *M* (SD)	Accept low *M* (SD)
Fz (μV; 500–1750 ms)	3.15 (5.21)	2.62 (5.09)	3.75 (5.34)	0.83 (4.45)
Cz (μV; 500–1750 ms)	8.51 (4.81)	7.19 (4.56)	7.97 (5.01)	4.90 (5.15)
Pz (μV; 500–1750 ms)	9.01 (3.84)	8.06 (4.74)	8.37 (4.28)	6.24 (4.49)
Fz (μV; 1750–3000 ms)	5.11 (6.14)	3.71 (6.34)	5.49 (6.32)	3.78 (4.85)
Cz (μV; 1750–3000 ms)	8.86 (5.55)	6.65 (5.26)	7.26 (5.76)	5.82 (6.03)
Pz (μV; 1750–3000 ms)	7.03 (4.75)	5.43 (5.42)	5.27 (5.41)	4.35 (5.27)
Valence	3.09 (0.89)	3.96 (0.63)	3.16 (0.93)	3.91 (0.68)
Arousal	4.10 (1.57)	2.82 (1.18)	4.06 (1.64)	2.76 (1.12)

*Note:* Valence and arousal were rated on the 1 to 9 SAM scales with higher numbers indicating more pleasant and arousing ratings. “Low” and “High” refer to low‐ and high‐arousing trials, respectively.

#### 3.2.1 LPP

##### 3.2.1.1 500–1750 ms

As expected, high‐arousing pictures elicited larger LPPs than low‐arousing pictures, *F*(1, 32) = 24.34, *p <* 0.001, *η*_*p*_^2^ = 0.43. There was also a main effect of condition, *F*(1, 32) = 7.80, *p =* 0.009, *η*_*p*_^2^ = 0.20 (Previously viewed > Previously accepted), which was qualified by a condition × arousal interaction, *F*(1, 32) = 4.81, *p* = 0.04, *η*_*p*_^2^ = 0.13. Follow‐up paired *t*‐tests revealed that previously accepted low‐arousing, *t*(32) = 3.68, *p* < 0.001, *d* = 0.64, but not high‐arousing (*p* = 0.74) pictures elicited smaller LPPs compared to previously viewed pictures.

##### 3.2.1.2 1750–3000 ms

As expected, high‐arousing pictures elicited larger LPPs than low‐arousing pictures, *F*(1, 32) = 7.34, *p* = 0.01, *η*_*p*_^2^ = 0.19. In addition, there was a significant condition × electrode interaction, *F*(1.96, 62.62) = 5.03, *p* = 0.01, *η*_*p*_^2^ = 0.14, such that pictures that had previously been presented on accept trials elicited smaller LPPs than those presented on view trials at Pz, *t*(32) = 2.46, *p* = 0.02, *d* = 0.43 (Cz, *p* = 0.09; Fz, *p* = 0.72). No other effects reach significance (*p*s > 0.14).

#### 3.2.2 Ratings

The main effect of arousal was significant for both valence, *F*(1,32) = 82.86, *p* < 0.001, *η*_*p*_^2^ = 0.72, and arousal ratings, *F*(1,32) = 141.92, *p* < 0.001, *η*_*p*_^2^ = 0.82, indicating that high‐arousing pictures were rated as more arousing and more unpleasant than low‐arousing pictures. No other effects reach significance for either valence (*p*s > 0.19) or arousal (*p*s > 0.47).

## 4 Discussion

The engagement‐disengagement framework may provide a useful way of understanding the relative strengths of emotion regulation strategies (MacNamara 2025). Acceptance has been understudied in the literature to‐date, leaving open questions about whether it behaves similarly to other engaged strategies. Here, we found that acceptance was only effective at reducing the electrocortical processing of low‐ and not high‐arousing pictures, Nonetheless, when the same pictures were encountered again without instructions to regulate, both low‐ and high‐arousing pictures that had previously been accepted elicited smaller LPPs than those that had been previously viewed—at least during the later portion of picture viewing. In addition, acceptance reduced arousal ratings for both low‐ and high‐arousing pictures during the first task, though effects did not persist to affect ratings in the second task. Results suggest that while acceptance is less effective at down‐regulating negative emotion in response to highly arousing stimuli in the short term, emotional engagement via acceptance gives way to longer‐term reductions in electrocortical responding to both low‐ and high‐arousing stimuli when stimuli are encountered again later on.

In prior work that did not control for picture arousal level, acceptance led to overall increases in the electrocortical processing of pictures (Boehme et al. 2019; Uusberg et al. 2016). In contrast, here we found that acceptance decreased the LPP, but only to low‐arousing pictures. One possible explanation for this discrepancy could be differences in how participants were instructed to engage in acceptance. That is, prior investigations centered on the present‐moment aspect of acceptance, drawing on principles of mindfulness (see Boehme et al. 2019; Uusberg et al. 2016). In so doing, prior work may have observed increases in the electrocortical processing of pictures because participants may have directed attention toward their emotional and bodily experiences while encountering negative stimuli. Here, we were interested in aspects of acceptance that go beyond awareness of thoughts and emotions. As such, our instructions to participants also emphasized that thoughts and emotions do not need to control or define an individual (Hayes et al. 1999). Therefore, participants may have engaged with negative stimulus content but may have also altered their perspective so that they refrained from focusing exclusively on this response. Given the heterogeneity of how acceptance is operationalized across studies, future work may wish to assess how different elements of acceptance modulate electrocortical and subjective response to stimuli (see Wojnarowska et al. 2020).

Overall, our findings fit within the broader literature on engaged emotion regulation strategies. Specifically, reappraisal is less effective and less preferred by participants when stimuli are highly arousing (Langeslag and Surti 2017; Shafir et al. 2015; Sheppes et al. 2011, 2014). On the other hand, we observed an effect of acceptance only in the early window of the LPP, whereas reappraisal is typically viewed as taking take time to implement, and takes effect more slowly than disengaged strategies (MacNamara 2025; Schönfelder et al. 2014; Thiruchselvam et al. 2011). One potential explanation could be differences in how reappraisal and acceptance are employed. To implement reappraisal, a person must first appraise, then successfully generate a reinterpretation of the situation, which can be time‐consuming. Conversely, acceptance does not necessitate that individuals think of an alternative interpretation for each stimulus, the effects of acceptance may have been able to take hold relatively early during picture presentation. Although given that acceptance did not reduce the LPP during the later part of picture presentation, the short‐term effects of acceptance may also be somewhat limited, at least at the electrocortical level. Taken together, these findings suggest that similar to other engaged strategies, acceptance may be better‐suited to less arousing stimuli (at least in the short term) but may exert faster yet shorter‐lived effects than other engaged strategies—that is, reappraisal.

Nonetheless, acceptance did result in reduced self‐reported arousal ratings for both low‐ and high‐arousing stimuli. This is in line with previous work examining the effect of arousal on the immediate efficacy of reappraisal. Specifically, Shafir et al. (2015) found that reappraisal reduced self‐reported negative emotion for both low‐ and high‐arousing pictures. Additionally, Langeslag and Surti (2017) also found that reappraisal (versus view) resulted in more pleasant ratings for both low‐ and high‐arousing pictures, even though it did not attenuate the LPP to low‐arousing pictures and increased LPP amplitudes to high‐arousing pictures. The present study extends these findings to acceptance, suggesting that although the efficacy of engaged strategies may be moderated by stimulus arousal on the electrocortical level, their effect on subjective ratings does not appear to be modulated by picture arousal level. This discrepancy could indicate that the LPP reflects differences in how low‐ and high‐arousing emotional stimuli are processed that occur outside of conscious awareness, and thus, are not reflected in self‐report ratings. Alternatively demand characteristics of the experiment could have influenced picture ratings but not the LPP. Overall, our results suggest that, like reappraisal, acceptance reduces subjective arousal ratings for both low‐ and high‐arousing stimuli.

Critically, acceptance's short‐term limitations appear to be offset by meaningful long‐term benefits. When participants were later exposed to the same pictures, low‐arousing pictures that had previously been accepted elicited smaller LPP amplitudes during the early part of picture presentation, and by the later part of picture presentation, this effect was evident for both low‐ and high‐arousing pictures. This finding is consistent with a broader literature suggesting that engaged emotion regulation strategies such as reappraisal support longer‐term reductions in the electrocortical processing of negative stimuli that are encountered later without instructions to down‐regulate (Ahn et al. 2015; Hermann et al. 2017, 2021; MacNamara 2025). Via engagement with negative emotions elicited by each picture, acceptance may have facilitated exposure and habituation, lessening emotional response upon re‐exposure (see Uusberg et al. 2016). Further, we found that acceptance led to faster reductions for previously seen low‐ versus high‐arousing pictures, which is in line with the broader literature suggesting engaged strategies may be easier to implement for low‐arousing stimuli (Langeslag and Surti 2017; Shafir et al. 2015). Nonetheless, though less effective for high‐arousing stimuli in the moment, engaged strategies like acceptance may facilitate longer‐term reductions in electrocortical responding to both low‐ and high‐arousing stimuli.

Regarding the distribution of the observed LPP effects in both tasks, the effect of acceptance was observed at frontocentral sites during active regulation, versus at more parietal sites during passive picture viewing. Prior work on acceptance has measured the LPP at centroparietal sites (Boehme et al. 2019; Uusberg et al. 2016). However in some studies, reappraisal has been found to lead to initial increases in a frontal LPP, particularly in response to highly arousing stimuli, which could reflect greater increases in effort associated with reappraisal's implementation for highly evocative picture content (Moser et al. 2014; Shafir et al. 2015). During the first task here, we found that acceptance led to decreases in the LPP to low‐arousing pictures at frontocentral sites. It is possible that the frontal distribution of these effects may reflect cognitive processes underlying the implementation of acceptance, while more parietal amplitudes in the second task may have reflected the fact that no active strategies were implemented.

Although acceptance did modulate the LPP during passive picture viewing, it did not affect self‐reported valence and arousal ratings in the second task. This is in contrast with prior work that has demonstrated engaged strategies, specifically reappraisal, can produce lasting changes in self‐report ratings, even without instructions to regulate (Ahn et al. 2015; Hermann et al. 2017, 2021; MacNamara et al. 2011). Though, some studies directly comparing the immediate effects of reappraisal and acceptance have found reappraisal may outperform acceptance in reducing self‐reported negative emotion to anxiety‐ and sadness‐inducing pictures (Boehme et al. 2019), as well as self‐negative beliefs (Goldin et al. 2019) and a disgust‐inducing stimuli (Wolgast et al. 2011). Overall, these prior results and those observed here suggest that some degree of caution is warranted, in that acceptance's focus on allowing emotions to exist without being overpowered by them may primarily affect neural responding via habituation, rather than lasting changes in subjective emotional experience when it is not actively being implemented.

Results of this study should be considered within its limitations. First, due to a programming error, data from the full study sample were not available for analysis from the passive picture viewing task. It is unclear whether EEG or self‐report findings would have differed for the second task if all data were available. Additionally, while stimuli presented in the lab are intended to simulate real‐world experiences that elicit negative emotion, these situations are often far more complex. Thus, it is unclear whether results observed in the lab would extend to real world contexts. Despite these limitations, the present study provides insight into the types of situations in which acceptance may be well‐suited to down‐regulate negative emotion. Future work along these lines may benefit from direct comparisons of how arousal may impact the short‐ and longer‐term effects of other engaged versus disengaged strategies to continue building out understanding of the engagement‐disengagement framework. Further, continued experimental research may wish to examine if acceptance shares other benefits of engaged strategies. For example, if its effects are able to generalize to similar but previously unseen stimuli.

In summary, results suggest acceptance is less effective in the short‐term for highly arousing stimuli though it brings about longer‐term reductions electrocortical responding to both low‐ and high‐arousing stimuli. These findings provide important insight into the relative strengths of acceptance, potentially informing when acceptance may be most effective at down‐regulating negative emotion based on desired immediate and lasting outcomes. Additionally, these results validate and extend the engagement‐disengagement framework by demonstrating acceptance appears to function similarly to other engaged strategies.

## Author Contributions

**Nicole R. Baughman:** formal analysis, writing – original draft. **Annmarie MacNamara:** conceptualization, writing – review and editing, supervision, methodology, funding acquisition.

## Funding

This work was supported in part by National Institute of Mental Health (NIMH) (Grant R01 MH125083 to A.M.).

## Conflicts of Interest

The authors declare no conflicts of interest.

## Supporting information


**Data S1:** psyp70410‐sup‐0001‐Supinfo.docx.

## Data Availability

The data that support the findings of this study are openly available in OSF at https://osf.io/zxgu4/overview.

## Endnotes

^1^
The following IAPS, EmoPics, and OASIS pictures were used. Picture numbers < 1000 are from the EmoPics image set and image numbers > 1000 are from the IAPS image set. Pictures with non‐numerical names are from the OASIS. *Low‐arousing*: 207, 218, 219, 220, 224, 228, 251, 325, 1050, 1930, 2110, 2141, 2301, 2700, 2710, 2900, 3180, 6020, 7380, 9140, 9185, 9220, 9340, 9415, 9421, 9429, 9430, 9445, 9560, 9571, 9600, 9830, 9908, Animal carcass 1, Bored pose 3, Car accident 4, Cat 7, Cockroach 1, Depressed pose 1, Depressed pose 2, Destruction 6, Fire 7, Miserable face 1, Miserable face 2, Sad face 8, Sad face 9, Sad pose 3, Scared face 4. *High‐arousing*: 209, 210, 222, 229, 239, 244, 249, 2095, 2205, 2345.1, 2375.1, 2799, 3101, 3160, 3181, 3185, 3220, 3230, 3280, 3301, 3500, 3530, 6520, 6563, 8230, 8231, 9042, 9050, 9163, 9183, 9187, 9250, 9254, 9301, 9302, 9322, 9412, 9413, 9428, 9435, 9584, 9900, 9903, 9921, Animal carcass 5, Injury 3, Shot 3, Tumor 1.
