# Supplementary material for: The Short‐Term Versus Long‐Term Effects of Acceptance and Moderation by Stimulus Arousal Level
Source: Psychophysiology. 2026 Sep 25;63(9):e70410. doi: 10.1111/psyp.70410 (PMC13614480; doi:10.1111/psyp.70410)
Supplement: Supplementary file 1 [file PSYP-63-e70410-s001.pdf]

The short-term versus long-term effects of acceptance

and moderation by stimulus arousal level

Supplementary Material

The following results were obtained for the Accept and View Task using only participants with data available for both tasks (n = 33). Overall, results were in line with those presented in the main manuscript.

LPP

Within this subsample, during the early time window, high-arousing pictures elicited larger LPP amplitudes than low-arousing pictures, $F(1, 32) = 28.19$, $p < .001$, $\eta_p^2 = .47$. Neither the main effect of condition ($p = .36$), nor the condition X arousal X electrode interaction ($p = .11$) reached significance. Within follow-up repeated measures ANOVAs, no significant effects involving condition emerged for high-arousing pictures (ps > .68). For low-arousing pictures, neither the main effect of condition ($p = .20$), nor the condition X electrode interaction ($p = .10$) reached significance. Paired t-tests revealed that the View > Accept difference trended toward significance at Cz ($p = .07$), but not Fz ($p = .25$) or Pz ($p = .61$), Notably, results at each electrode were in the same direction as the full sample (View > Accept). Additionally, only a main effect of arousal emerged during the late time window $F(1, 32) = 11.55$, $p = .002$, $\eta_p^2 = .27$, indicating greater amplitudes in response to high-arousing pictures.

Ratings

Participants rated pictures on accept trials as less arousing compared to view trials, $F(1, 32) = 7.51$, $p = .01$, $\eta_p^2 = .19$. Additionally, high-arousing pictures were rated as more arousing than low-arousing pictures, $F(1, 32) = 159.67$, $p < .001$, $\eta_p^2 = .82$. The arousal X condition interaction did not reach significance for arousal ratings ($p = .75$). Pictures presented on accept trials were rated as more pleasant, $F(1, 32) = 4.49$, $p = .04$, $\eta_p^2 = .12$, than those presented on view trials. High-arousing pictures were rated as less pleasant that low-arousing pictures, $F(1, 32) = 79.05$, $p < .001$, $\eta_p^2 = .71$. The arousal X condition interaction was not significant for valence ratings (p = .28).
